# Postpartum Psychosis after Traumatic Cesarean Delivery

**DOI:** 10.3390/healthcare9050588

**Published:** 2021-05-16

**Authors:** Evangelia Antoniou, Eirini Orovou, Kassiani Politou, Alexandros Papatrechas, Ermioni Palaska, Angeliki Sarella, Maria Dagla

**Affiliations:** 1 Department of Midwifery, University of West Attica, 12243 Athens, Greece; eorovou@uniwa.gr (E.O.); epalaska@uniwa.gr (E.P.); asare@uniwa.gr (A.S.); mariadagla@uniwa.gr (M.D.); 2 Non-Profit/Non Governmental Organization (NGO) “Fainareti”, 12243 Athens, Greece; k.politou@yahoo.co.uk (K.P.); al_papa66@hotmail.com (A.P.)

**Keywords:** postpartum psychosis case report, bipolar disorder, emergency cesarean section, traumatic childbirth, acute delirium episode after delivery

## Abstract

An emergency cesarean delivery can be a traumatic childbirth experience for a woman and a risk factor for postpartum psychosis, especially in a patient with a history of bipolar disorder. This article describes the case of a pregnant woman with an unknown history of bipolar disorder who developed an acute psychotic reaction during the procedure of an emergency caesarian section and switched to mania. The purpose of this case study is for perinatal health care professionals to identify suspicious symptoms and promptly refer to psychiatric services so as to ensure the mother’s and the newborn’s safety. This case study highlights the importance of assessing women with bipolar disorder or a previous psychotic episode for the risk of psychiatric complications in pregnancy and after childbirth. Midwifery education on perinatal mental health is crucial for the detection of suspicious symptoms and early referral to a specialist.

## 1. Introduction

Postpartum psychosis (PP) is a rare illness compared to the rates of postpartum anxiety and depression. It occurs in 0.89 to 2.6 out of every 1000 births. Postpartum psychosis has a rapid onset shortly after childbirth [1]. PP symptoms can include difficulty in communication, rapid mood swings, paranoia and suspiciousness, delusions or hallucinations, and hyperactivity [2]. The onset of most postpartum psychosis episodes is within 2 weeks of delivery [3]. These symptoms develop quickly and dramatically and in the majority of cases require hospitalization [4].

The most significant risk factors for postpartum psychosis are a previous psychotic episode and a family or personal history of bipolar disorder (BD) or PP [2,5]. A previous study suggested that up to 95% of women with PP fulfilled the diagnostic criteria for cyclic mood disorders at a 5-year follow-up [6,7]. Other risk factors for PP include primiparity [8,9], the extremes of reproductive age, cesarean section [10,11], sleep deprivation [12], withdrawal of mood stabilizing medication [13], poor socio-economic status, and postpartum maternal and neonatal complications [10]. Women with postpartum complications were twice as likely to have PP compared to those without complications, and the offspring of women with PP who had been hospitalized were four times more likely to die within the first year after birth [14]. Recent studies added weight to an association between preeclampsia and postpartum psychiatric episodes, which was probably due to the psychological impact of the disease but also to the vascular inflammatory condition caused by preeclampsia [9]. Historically, other cerebral or systemic conditions, such as eclampsia, delirium, thyroid disorders, or infection were important causes of psychosis occurring at that time [15].

BDs, including Bipolar I, Bipolar II, and Cyclothymic disorder, are chronic psychiatric disorders characterized by alternating episodes of major depression and mania or hypomania, or mixtures of manic and depressive elements [16]. In a large epidemiological study involving 11 countries, the overall prevalence of the bipolar disorder spectrum was found to be 2.4% with a prevalence of 0.6% of BD I and 0.4% for BD II [17]. As for the gender, there is no difference in the prevalence of all types of BD among men and women [18]. Τhe disorder typically occurs between the period of late adolescence to early adulthood, placing women at sevenfold risk for morbidity, disability, and mortality throughout reproductive years [19,20].

Furthermore, women with a family history of postpartum recurrence run a higher risk of recurrence in this period [21]. Several studies reveal depressive or manic symptoms in up to 70% of pregnant women with BD [22,23]. The risk is higher if a woman with bipolar disorder has discontinued her medication during pregnancy, which is something commonly observed. The severity of PP is underscored by the fact that in rare but tragic cases, the illness can lead to suicide and child protection issues. Although suicide is rare, it is one of the leading causes of maternal death [15,24]. Infrequently, in cases of severe psychosis or psychotic depression, infanticide may also be committed [25]. The postpartum period also increases seventyfold the risk of suicide, which can be a cause of maternal death in two out of 1000 women [26], while >33% had recurrent PP, and in 5%, the condition was established in the postpartum period [27]. 

The significant impact of BD on the type of delivery has been reported in a case-control study published in 2019. In this research, women with BD run a twofold increased risk for cesarean delivery compared to women without BD, of similar age and parity. An explanation for this phenomenon could be a personal history of BD and the comorbidity of these patients [28].

## 2. Case Presentation Section

### 2.1. Initial Presentation and Consent

This case study is part of a larger survey on postpartum PTSD [29]. Due to the rarity of the incident, we proceeded after the written consent of the patient for further investigation. The purpose of this study was to raise the awareness of obstetricians and midwives in the follow-up of suspicious signs during antenatal care that could develop into psychiatric emergencies during or after delivery. 

In this case report, we describe the episode of postpartum psychosis of a primiparus woman in her early 30s, with undiagnosed bipolar disorder who was admitted for an emergency cesarean section due to preeclampsia. The patient was closely psychiatrically observed, and the psychiatric team soon reached the diagnosis of postpartum psychosis. She was started on treatment “with olanzapine 10 mg, diazepam 10 mg, and biperiden 4 mg per os”, to control symptoms including labile mood with racing thoughts and disorganized behavior, decreased need for sleep, gradual onset of exaggerated sense of well-being, and grandiose behavior.

### 2.2. Labor and Delivery

The patient presented to the maternity hospital due to the onset of full-term labor contractions. She was very anxious and labile in her mood, talking loudly in the waiting room. She was examined by the gynecologist and was diagnosed with preeclampsia (200/100 mm Hg). The medical team immediately decided to proceed with a cesarean section within an hour after her admittance to the hospital. As reported by the doctors, she quickly became paranoid with the persecutory thought that the doctors “wanted to steal her baby”. She became irritable and argumentative and that made it difficult to cooperate with the staff.

According to her medical file and the records from the maternity ward, when the patient was taken to the operating room, there was an exacerbation of her subjective fears of threat; she became even more stimulated and aggressive with the staff, threw and broke materials, and shouted loudly that they wanted to steal her baby. She expressed the delusional belief that a specific woman, her father’s current wife, made them do that to take revenge, while doctors, midwives, and other staff members were considered as part of this conspiracy led by this jealous woman. The on-duty psychiatrist was immediately called; the patient underwent an emergency cesarean section under general anesthesia. The newborn, a 3500gr girl, was removed from her mother for safety precautions.

### 2.3. Postpartum Presentation

On postpartum day 2, the patient was depressed and described moments of anxiety, loss of control, and a feeling of imminent death for her and her baby. She reported intense terror at the sight of the operating room because she was prepared for a natural birth. She had a vague memory of her behavior and that she spoke abruptly to doctors and midwives, but her memories went as far as the moment the vein catheter entered her hand. At that point, she was no longer preoccupied with the “jealous woman”. She was rather confused and feeling guilty for what she had caused when the facts were described to her by her family, and she reported that this was something out of her character. She also reported feeling guilty and a failure for not being able to have a natural delivery. It had been a desired pregnancy and she had been looking forward to the birth of her child. During the last two pregnancy trimesters, she had gained 40 kilos (from 55–95 kg), which resulted in preeclampsia, increased follow-up sessions, and intense anxiety.

The psychiatric episode was described by the psychiatrist as an acute manic episode, and during hospitalization, diazepam was administered intramuscularly to control the symptoms. During hospitalization, a neurological and nephrological evaluation followed. After the cesarean section, there was no urine albumen, and the values of blood pressure were normal. The results of further tests of electrolytes, calcium, uric acid, phosphorus, and TSH levels were within normal limits.

### 2.4. Follow-Up 

The patient completed assessments by interview at three time-points: (a) Day 2 postpartum; (b) Week 6 postpartum; and (c) Week 10 postpartum. For the purpose of the study, several questionnaires were administered. The Medical/Demographic Questionnaire was administered to assess maternal, neonatal, mental, and social characteristics of the postpartum woman. The Life Events Checklist (LEC) from the National Center for PTSD [30] was administered to assess traumatic events in a person’s lifetime known to result in PTSD. The Stressor Perinatal Criterion detects the mother’s stress before, during, and after a cesarean section including questions about the sense of loss of her and her baby’s life [31]. The Posttraumatic Checklist-5 (PCL-5) [32] measures PTSD symptoms. The postpartum woman met all PTSD criteria according to PCL-5, but she was under treatment with psychotropic medications.

Day 2 postpartum: After completing the LEC-5, which included only the death of her mother, she completed the Stressor Perinatal Criterion, which indicated the direct exposure of mother and neonate to a threatened death during the cesarean section. At this stage, the patient completed the Medical/Demographic Questionnaire, which included much of her medical and psychosocial history.

During the interview, the patient was anxious, her speech was slow and pressured, and the communication with her was difficult. Although she accepted her participation in the study, during the interview, she did not want to have eye contact with the interviewer, and she chose to cover her face with a sheet.

Week 6 Postpartum: In week 6 postpartum, after completing the PCL-5, the patient appeared wellkept, with heavy make-up, was very friendly and talkative while easily distracted. She reported that she liked going out with friends and believed that her daughter and herself have excellent abilities and strengths (i.e., she believed that there were magical substances in her and in her baby’s blood). She did not worry about the extra weight; she believed that her mother, who was obese, was alive again in her body throughout her pregnancy. Her speech was fast and her thoughts moved quickly from one theme to another. She referred many times to the fear that the episode would happen again or that her condition would become chronic, which was especially concerning for her. 

Week 10 Postpartum: In week 10 postpartum, the patient described her mood as “excellent”. Although she was not back to her baseline according to the psychiatric assessment, she had gained some insight into her condition and diagnosis, was able to recognize the consequences and meaning of her mood changes as well as the basic other symptoms of bipolar disorder. She started to refer to feelings of guilt for not being able to be well enough and stay more attuned to her baby until that time.

### 2.5. Past History

According to the patient’s previous psychiatric history, it was only after the episode of postpartum psychosis that the medical team collected more information from her family. The patient reported a brief psychotic episode as an adolescent after her mother’s death. At that time, she exhibited agitated and aggressive behavior with a persecutory delusion that her father’s mistress had killed her mother by intravenously administering poison. While hospitalization was recommended, the family refused, and she only received treatment with olanzapine for 6 months. When the patient was asked to describe the incident, she simply said that she did some “crazy things” because she had lost her beloved mother. With respect to her personal history, the patient was the eldest daughter of a low-income family. She was born and raised in the countryside, and she had a difficult childhood. During the interview, she said that she had been married for a few years. It was an arranged marriage, and her husband was away for long periods of time because of his work; however, she had enough practical support by her mother-in-law. Her personal health history was uncomplicated. 

Regarding her family history, in the first and second interview, she mentioned that her mother might have suffered from depression. However, during the third interview (week 10 postpartum), the patient felt safe enough to reveal that her mother had a history of bipolar disorder and apologized for not saying that in the first place because she did not want to be stigmatized.

## 3. Recommendations to Midwives

Not only the perinatal mental health services but also the perinatal care health professionals can contribute to the prevention and timely diagnosis of the mental disorders. Midwives, as members of the perinatal mental health team, provide the family with education, emotional support and security, and a safe risk assessment. Regarding treatment, midwives can educate women about the expected side effects of pharmacotherapy and the importance of treatment adherence [33]. Midwifery care for women with PP can be the connecting link between the obstetric and mental health system. A woman’s relationship with health care practitioners is critical for improving psychosocial functioning and preventing psychiatric relapse [34]. According to Pembroke [35], many midwives have a professional ability that allows them to share their responsibility, availability, support, and respect to other women. Appropriate identification and psychoeducation of the patient with severe mental illness or at high risk of developing such an illness during pregnancy or postpartum is of great importance. For this special population group of women in the perinatal period, psychotherapy and psychosocial interventions precede, regardless of the need for medication. Midwives will be the first to recognize the symptoms, determine their consulting role in the treatment, participate in the perinatal mental health team, and facilitate the improvement and recuperation of patients with PP. 

More specifically, during the prenatal period, midwives can play a critical role in identifying risk factors for PP, such as the history of BD or a family history of mental health illness. Midwives provide emotional support, guidance, and empowerment to pregnant women with increased risk factors for PP, preparing them for a non-traumatic delivery that could trigger postpartum mental disorder.

## 4. Discussion 

This patient is a typical example of the effects that follow a patient with untreated BD in the postpartum period. It is very common for women having BD to experience PP, while prompt treatment is necessary to get PP under control. 

PP is an emergency condition that requires immediate psychiatric evaluation. The symptoms of PP can be very frightening for women who are affected, for families and midwives. Women with PP need to be evaluated for underlying organic medical conditions, which can cause psychiatric-like symptoms, such as diabetic ketoacidosis, Graves’s disease, stroke, meningitis, preeclampsia, and adverse effects from medications [36,37,38]. As PP includes delusional beliefs, disorganized behavior, and cognitive disturbance, it must be differentiated from other postpartum mental disorders. 

A key aspect of the care plan should be an individualized risk–benefit analysis around medication. The evidence of response to particular medication as well as the dosage of acute episode and maintenance treatment are also important considerations [39,40,41]. Admission in a mother and baby unit might be necessary, even for women with supportive families [42].

The treatment of women with BD during pregnancy is one of the greatest challenges in psychiatry, because the goal is to prevent fetal toxicity and maintain maternal well-being [20]. The patients with BD must be under the supervision of mental health services during pregnancy. When taking the medical history, it is important to ask the pregnant woman (a) for any previous mental disorder history, including previous perinatal mental health difficulties; (b) history of any pharmaceutical treatment in a previous or this phase; (c) prior hospitalizations; (d) family history of perinatal mental disorders [39]. Stable patients may stop treatment with mood stabilizers before conception, but we must keep in mind that the termination of maintenance treatment is associated with high rates of relapse, especially if said termination is abrupt [22,43]. According to Cohen, 1995 [44], the risk of relapse of patients that did not receive psychoprophylactic treatment was 8.6 times higher vs. women receiving treatment.

A mother suffering from BD may neglect her health and self-care, and she may not be able to sufficiently respond to her baby’s needs [45]. Regarding the risk for mental disorders in the perinatal period, suicide, although rare, has been found to be the most frequent reason of death in the perinatal period [46], while most cases of suicide are committed after labor. Approximately half of the women that committed suicide had some prior history of a major depressive disorder [47].

## 5. Conclusions

This is a case report of a woman with BD, with insufficient mental health care before and during her pregnancy, who underwent a Cesarean section, which resulted in a severe psychotic episode postpartum, with detrimental consequences for the mother and the mother–infant relationship. Since large surveys on PP are rare, this case study highlights the need for prenatal psychoeducation of women, especially those with a history of mental disorders. The need for continuing midwifery education on perinatal mental health issues is highlighted by this case.

## Data Availability

Data sharing not applicable.
